# Spatial Separation of Plk1 Phosphorylation and Activity

**DOI:** 10.3389/fonc.2015.00132

**Published:** 2015-06-10

**Authors:** Wytse Bruinsma, Melinda Aprelia, Jolanda Kool, Libor Macurek, Arne Lindqvist, René H. Medema

**Affiliations:** ^1^Department of Cell Biology, The Netherlands Cancer Institute, Amsterdam, Netherlands; ^2^Department of Medical Oncology and Cancer Genomics Center, University Medical Center Utrecht, Utrecht, Netherlands; ^3^Laboratory of Cancer Cell Biology, Institute of Molecular Genetics of the ASCR, v. v. i., Prague, Czech Republic; ^4^Department of Cell and Molecular Biology, Karolinska Institute, Stockholm, Sweden

**Keywords:** plk1, aurora kinase, cell cycle, checkpoint recovery, bora

## Abstract

Polo-like kinase 1 (Plk1) is one of the major kinases controlling mitosis and cell division. Plk1 is first recruited to the centrosome in S phase, then appears on the kinetochores in late G2, and at the end of mitosis, it translocates to the central spindle. Activation of Plk1 requires phosphorylation of T210 by Aurora A, an event that critically depends on the co-factor Bora. However, conflicting reports exist as to where Plk1 is first activated. Phosphorylation of T210 is first observed at the centrosomes, but kinase activity seems to be restricted to the nucleus in the earlier phases of G2. Here, we demonstrate that Plk1 activity manifests itself first in the nucleus using a nuclear FRET-based biosensor for Plk1 activity. However, we find that Bora is restricted to the cytoplasm and that Plk1 is phosphorylated on T210 at the centrosomes. Our data demonstrate that while Plk1 activation occurs on centrosomes, downstream target phosphorylation by Plk1 first occurs in the nucleus. We discuss several explanations for this surprising separation of activation and function.

## Introduction

Polo-like kinase 1 (Plk1) is an important kinase during the cell cycle. It controls several key processes that drive cells into and through mitosis such as centrosome maturation, spindle assembly, sister chromatid cohesion, cytokinesis, and recovery from a DNA damage-induced arrest (1). To carry out these specific functions, Plk1 is recruited to very specific subcellular sites throughout the cell cycle. Plk1 is predominantly localized at the centrosomes during S-phase, G2, and mitosis. In addition, Plk1 localizes to the kinetochores during G2 and mitosis and at the end of mitosis when the chromosomes segregate Plk1 translocates to the spindle midzone (2). This localization of Plk1 depends on its Polo-box domain that can efficiently bind to pre-phosphorylated substrates (3, 4) and can target Plk1 to its various subcellular localizations in the cell (5–9). Plk1 is first activated in G2, by phosphorylation on its T210-residue (10). The Aurora A kinase is responsible for this phosphorylation event and requires binding of the co-factor Bora in order to be able to phosphorylate Plk1 (11, 12). The notion that Plk1 is activated in G2 is well established; however, the exact location in the cell where Aurora A phosphorylates Plk1 is not clear. Both proteins localize to centrosomes and T210-phosphorylated Plk1 has also been observed at centrosomes (1, 12, 13). However, antibodies targeting T210-phosphorylated Plk1 have been shown to recognize off-target epitopes, making analysis based on these signals ambiguous (14). In addition, measuring Plk1 activity in living cells using a FRET-based biosensor specifically regulated by Plk1 showed that substrate phosphorylation by Plk1 activity is first observed in the nucleus at around 5 h before mitosis, when S-phase is completed, with activity spreading to the cytoplasm approximately 2 h before mitosis (12, 15). A straightforward interpretation of this observation is complicated by the fact that the FRET-based biosensor is diffusible and active transportation of the FRET-based biosensor could contribute to the observed effects. Moreover, the localization of the co-factor Bora is not extensively studied. In *Drosophila*, where Bora was first identified as a co-factor for Aurora A, it was shown that Bora is located in the nucleus and translocates to the cytoplasm in early prophase (16). However, localization of Bora during the cell cycle in human cells seems to be regulated differently, although this is mainly based on exogenous Bora (17).

Here we show, using a nuclear localized FRET-based biosensor, that initial substrate phosphorylation by Plk1 in the nucleus is not the result of a diffusing FRET-probe, but that substrate phosphorylation by Plk1 initially occurs in the nucleus. However, we find that sequestration of Plk1 in the nucleus prevents phosphorylation of T210. This is in contrast to sequestration of Plk1 to the centrosomes, where Plk1 can get phosphorylated on T210. Thus, our data show that Plk1 is phosphorylated and activated at the centrosome, but Plk1 activity is first seen to rise in the nucleus. We were unable to reconstitute Plk1 function by mutants of Plk1 that strictly localized at the centrosomes or in the nucleus, indicating that Plk1 needs to be able to diffuse from the centrosomes to the nucleus in order to be fully functional. Finally, we show that Bora localizes strictly in the cytoplasm in human cells and Bora degradation is induced approximately 2 h before cells enter mitosis. This degradation does not completely remove all Bora, as we have shown previously, and Bora/Aurora A continue to activate Plk1 also in mitosis (14, 17). Taken together, our data show that Plk1 is activated in the cytoplasm where both Bora and Aurora A are localized; however, translocation of Plk1 to the nucleus seems to be required for the establishment of target phosphorylation, as it is where Plk1 activity first appears.

## Materials and Methods

### Cell culture, antibodies, and reagents

Human osteosarcoma U2OS cells were grown in Dulbecco’s modified Eagle’s medium (DMEM, Gibco) supplemented with 6% FCS (Lonza), 2 mM l-glutamine, 100 U/ml penicillin, and 100 mg/ml streptomycin. Cell lines expressing LAP-Plk1, AKAP-LAP-Plk1, H2B-LAP-Plk1, and GFP-Bora under the control of tetracycline-inducible were cultured in DMEM containing Tet system approved fetal bovine serum (Lonza). Antibodies that were used were directed against Plk1 (18, 19), Plk1, Cyclin B1, Actin (all from Santa Cruz), GFP (Roche), Plk1-pT210 (BD), Tubulin (Sigma), Bora (17), Aurora A (Cell Signaling) Histone H3-pS10, and H2AX (both from Upstate). The following drugs were used: BI 2536 (100 nM, Boehringer Ingelheim Pharma), MLN8054 (1 μM, Millennium Pharmaceuticals), thymidine (2.5 mM, Sigma), caffeine (5 mM, Sigma), adriamycin (0.5 μM, Sigma), nocodazole (250 ng/ml, Sigma), PI (Sigma) puromycin (Sigma, 2 μg/ml), and tetracyclin (Sigma, 1 μg/ml).

### Cloning and generation of stable cell lines

H2B-tagged versions of LAP-Plk1 and the FRET-based biosensor were generated in the following manner: H2B was amplified by PCR using the forward primer 5′-AAGCTTATGCCAGAGCCAGCGAAGTC-3′ and the reverse primer 5′-AAGCTTAGATCCTTAGCGCTGGTGTACTTGG-3′ and ligated into either the FRET-based biosensor or the LAP-Plk1 construct using the restriction enzyme *Hin*dIII (NEB). AKAP-LAP-Plk1 was generated in the following manner: the AKAP centrosomal binding doamina was amplified by PCR using the forward primer 5′-AAGCTTGCCACCATGGCCAACATTGAAGCC-3′ and the reverse primer 5′-CTTAAGCTTCTCATGCCAGCATGAAATTG-3′ and ligated into the LAP-Plk1 consruct using the restriction enzymes *Hin*dIII and *Eco*RI (NEB). The pTON-GFP-Bora construct has been described previously (12). U2OS-derived U2TR cells stably expressing LAP-Plk1 have been described previously (12). U2TR cells stably expressing AKAP-LAP-Plk1, H2B-LAP-Plk1, and GFP-Bora were generated by calcium phosphate transfection of the constructs, selection of stable clones by zeocin (400 mg/ml, Invitrogen) treatment for 2 weeks followed by clonal selection. Stable clones were grown in media containing tetracycline system approved fetal bovine serum (Lonza). To induce expression, cells were treated for indicated times with tetracycline (1 mg/ml).

### Transfections, cell synchronization, and FACS

Cells were transfected using calcium phosphate transfection of plasmids. For selection of transfected cells with pSuper constructs, GFP-spectrin was co-transfected for FACS or with pBABE-puro followed by puromycin treatment for western blot analysis. For analysis of checkpoint recovery, cells were synchronized at the G1/S-border by thymidine (2.5 mM) for 24 h followed by a 6 h release and 1 h incubation with Adriamycin (0.5 μM). Afterwards, cells were kept for 16 h in nocodazole (250 ng/ml). Recovery was induced by adding caffeine (5 mM). Unperturbed mitotic entry was assayed by a 24 h thymidine block followed by a release into nocodazole. For reconstitution assays, expression was induced by addition of tetracycline (1 mg/ml) at the indicated times. For FACS analysis, cells were transfected were harvested by trypsinization and fixed with ice-cold 70% ethanol. Cells were stained using the anti-Histone H3-pS10 antibodies (Millipore) and Alexa488-conjugated secondary antibodies (Molecular Probes). DNA was stained using propidium iodide and samples were analyzed on a FACSCalibur flow cytometer (BD biosciences). Cell cycle distribution was determined by flow cytometry counting 10^4^ events of cells positive for GFP-spectrin.

### FRET- and live cell imaging

For time-lapse microscopy, cells were grown on LabTek II chambered coverglasses in Leibovitz’s L-15 medium (Gibco) supplemented with 6% FCS (Lonza), 2 mM l-glutamine, 100 U/ml penicillin, and 100 mg/ml streptomycin, and were imaged with DIC on a Zeiss Axiovert 200M using 20 × 0.75NA objectives or on a Deltavision imaging system using 20 × 0.75NA objectives. Images were taken every 20 min. GFP-Bora levels were quantified by measuring the integrated density of the GFP signal in cells. Background was subtracted using an area that contained no cells. The FRET-based biosensor for monitoring PLK1 activity has been described previously (12, 20). The CFP/YFP emission ratio after CFP excitation of U2OS cells stably expressing the FRET-based biosensor was monitored on a Deltavision Elite imaging system, using a 20 × 0.75NA objective. Images were acquired every 20 min. The images were processed with ImageJ using the Ratio Plus plug-in (http://rsb.info.nih.gov/ij/).

### Cellular fractionation immunoprecipitations and western blotting

Chromatin fractionation was performed as described (21). Soluble cytosolic proteins were extracted from U2OS cells by incubating cells in buffer A (10 mm HEPES, pH 7.9, 10 mm KCl, 1.5 mm MgCl2, 0.34M sucrose, 10% glycerol, 1 mm DTT, 0.1% Triton X-100, and protease inhibitor cocktail) at 4°C for 10 min and spinning down at 1500 × *g* for 2 min. Soluble nuclear fraction was obtained by extraction of pelleted nuclei with an equal amount of buffer B (10 mm HEPES, pH 7.9, 3 mm EDTA, 0.2 mm EGTA, 1 mm DTT) and spinning down at 2000 × *g* for 2 min. Insoluble chromatin was washed with buffer B and finally resuspended in SDS sample buffer. For immunoprecipitations, cells were lysed in with 1 ml lysis buffer (1% NP-40, 50 mM Hepes pH 7.4, 150 mM NaCl, 1 mM EGTA, 1 mM MgCl2, 1 mM NaF, 1 mM Na3VO4, 25 mM β-glycerophophatese, 1 tablet of complete EDTA-free per 50 ml) on ice for 10 min. The lysate was cleared by centrifugation and 10% of supernatant was used for whole cell lysate. Immunoprecipitations were performed using S-protein beads (Novagen) or with antibodies bound to Dynabeads Protein A (Life Technologies). Beads were washed with TBST and incubated with the rest of the lysate at 4°C for 24 h. Beads were washed extensively with lysis buffer, after which bound protein was eluted with Laemmli sample buffer.

## Results

### The Plk1 FRET-probe is phosphorylated in the nucleus

**Figure 1:**
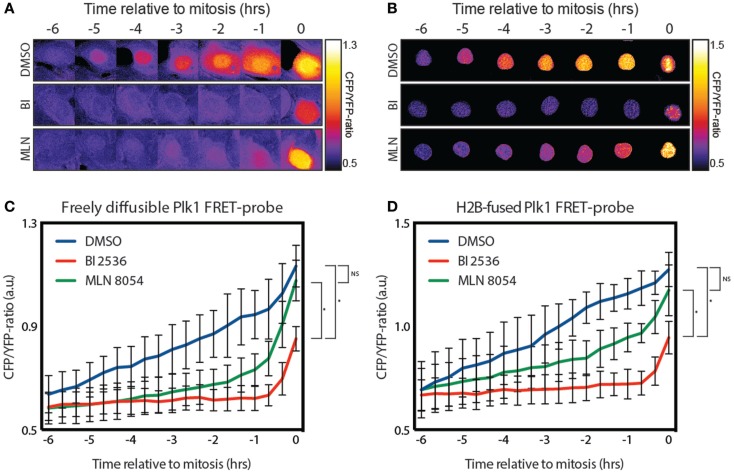
**Plk1 activity is first seen in the nucleus**. **(A)** Stills from a movie showing false color-coded CFP/YFP emission ratios. The stills show control-, BI 2536-, and MLN 8054-treated U2OS cells expressing the diffusible FRET-based biosensor for Plk1 activity while entering mitosis. BI 2536 was used at a concentration of 100 nM; MLN 8054 at a concentration of 1 μM. **(B)** Stills from a movie showing false color-coded CFP/YFP emission ratios of U2OS cells expressing the H2B-tagged FRET based biosensor were treated as in **(A)**. **(C)** Quantification of CFP/YFP-ratio of cells shown in **(A)**. Error bars represent the SD of 10 individual cells. **(D)** Quantification of CFP/YFP-ratio of cells shown in **(B)**. Error bars represent the SD of 10 individual cells; *p < 0.0001.

Plk1 localizes both at the centrosomes and kinetochores during G2. While most of the literature suggests that Plk1 is activated at the centrosomes, this is mainly based on immunofluoresence using anti-phospho-T210 antibodies (12), and the fact that Aurora A is localized at the centrosomes in G2 (13). However, using a FRET-based biosensor to measure the Plk1 activity in real-time, we found that Plk1 activation is first visible in the nucleus approximately 5 h before cells entered mitosis [Figure 1A; (12)]. Since this probe is not tethered, we wondered whether shuttling of an activated probe between the cytoplasm and the nucleus might account for this observation. To test this, we generated an H2B-tagged FRET-based biosensor for Plk1 that is localized exclusively in the nucleus (Figure 1B). We observed that cells expressing this construct entered mitosis normally and displayed similar kinetics of Plk1 activation in the nucleus (Figures 1C,D). In addition, pharmacological inhibition of Plk1 with the small molecule inhibitor BI 2536 (22) led in both cases to inhibition of Plk1 activity, except for a small signal in mitosis (Figures 1A–D), which we have previously shown to be dependent on the mitotic kinase Mps1 (14). In addition, inhibition of Aurora A with the small molecule inhibitor MLN 8054 (23) also led to similar kinetics of Plk1 activation where the initial activation is repressed (Figures 1A–D). Activation of Plk1 during the later stages of G2 is also dependent on Aurora A, but inhibition of Aurora A through MLN 8054 is not penetrant enough to achieve complete inhibition of its activity, as we have shown previously (14). Taken together, these results show that immobilization of the FRET-based biosensor for Plk1 does not affect the timing of its phosphorylation, demonstrating that substrate phosphorylation by Plk1 first becomes apparent in the nucleus, approximately 5 h before cells enter mitosis.

### Dynamic localization of Plk1 is important for checkpoint recovery

**Figure 2:**
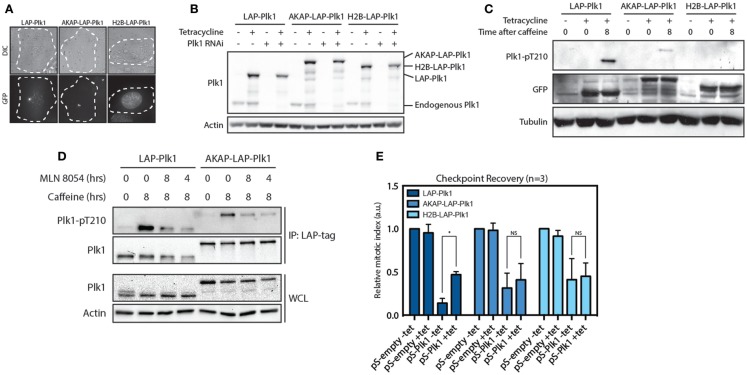
**Plk1 is activated at the centrosomes but needs to be dynamically localized**. **(A)** Expression of RNAi resistant LAP-Plk1, AKAP-LAP-Plk1 or H2B-LAP-Plk1 was induced in U2TR stably expressing these constructs by addition of tetracycline. DIC- and GFP-images were taken of representative cells. **(B)** Tetracycline inducible U2TR cells stably expressing RNAi resistant LAP-Plk1, AKAP-LAP-Plk1 or H2B-LAP-Plk1 were transfected with an empty pSuper, or a pSuper targeting endogenous Plk1. Cells were synchronized in G2 and damaged with 0.5 μM adriamycin for 1 h and expression was induced where indicated using tetracycline. 16 h after induction of DNA damage cell were harvested and analyzed by western blotting. **(C)** Tetracycline inducible U2TR cells stably expressing RNAi resistant LAP-Plk1, AKAP-LAP-Plk1 or H2B-LAP-Plk1 were synchronized in G2 and damaged with 0.5 μM adriamycin for 1 h and expression was induced where indicated using tetracycline for 16 h. Cells were harvested at the indicated time points after addition of caffeine and analyzed by western blotting. **(D)** Cells were treated as in C. MLN 8054 was added at a concentration of 1 μM either for 8 h together with caffeine or during the last 4 h of caffeine. LAP-tagged proteins were immunoprecipitated with S-protein agarose beads and analyzed by western blot. **(E)** Tetracycline inducible U2TR cells stably expressing RNAi resistant LAP-Plk1, AKAP-LAP-Plk1 or H2B-LAP-Plk1 were transfected with an empty pSuper, or a pSuper targeting endogenous Plk1. Cells were synchronized in G2 and damaged with 0.5 μM adriamycin for 1 h and expression was induced where indicated using tetracycline. Cells were arrested for 16 h, recovery was induced by caffeine addition for 8 h and the mitotic index was determined, based on the percentage of Histone H3-pS10 positive cells, using FACS. Error bars represent the SD of three independent experiments; *P < 0.001.

Since substrate phosphorylation by Plk1 is first observed in the nucleus during G2, we next asked if Plk1 needs to be on the centrosomes or in the nucleus in order to promote entry into mitosis during recovery from a DNA damage-induced arrest. To this end, we generated stable cell lines expressing tetracycline-inducible and RNAi-resistant variants of Plk1 that were either freely diffusible, or exclusively localized at centrosome or in the nucleus. For this purpose, we used EGFP-TEV-S (LAP)-tagged wild-type Plk1 (12, 24), an AKAP-LAP-Plk1 fusion that is physically tethered to the centrosome through fusion to the centrosomal targeting domain of AKAP450 (25) and an H2B-LAP-Plk1 fusion in which Plk1 is fused to H2B and is therefore located exclusively in the nucleus (Figure 2A). Using a short hairpin that targets endogenous Plk1 (26), we depleted the endogenous protein and used tetracycline-induced expression of the exogenous proteins for protein replacement (Figure 2B). We first analyzed if these fusion proteins could be phosphorylated at T210 during checkpoint recovery. To this end, we synchronized the cells in G2 and induced DNA damage by Adriamycin. As a consequence of checkpoint activation, these cells remain arrested in G2, and we subsequently induced checkpoint recovery by adding caffeine for 8 h. During this time, Plk1 gets activated through phosphorylation of T210 and cells resume the cell cycle and enter mitosis (12, 26). Indeed, LAP-Plk1 was efficiently phosphorylated at T210 after the addition of caffeine (Figure 2C). In accordance with the hypothesis that Plk1 is activated at centrosomes, we found that AKAP-LAP-Plk1 was also phosphorylated at T210. Although the phosphorylation of AKAP-LAP-Plk1 was less prominent than LAP-Plk1, we could not detect any phosphorylation of T210 on H2B-LAP-Plk1, suggesting that activation of Plk1 occurs outside of the nucleus (Figure 2C). Furthermore, inhibition of Aurora A affected phosphorylation of Plk1 at T210 in both LAP-Plk1 as well as LAP-AKAP-Plk1 showing that this phosphorylation is dependent on Aurora A (Figure 2D). Next, we wondered if the centrosomal- and nuclear-tethered Plk1 versions could induce checkpoint recovery, a well-established function of Plk1 (12, 26). To this end, we depleted endogenous Plk1 by RNAi and reconstituted Plk1 expression with the exogenous proteins prior to induction of recovery. We subsequently determined the amount of mitotic cells after 8 h of caffeine treatment as a measure of checkpoint recovery. Expression of the exogenous versions of Plk1 did not affect recovery in the presence of the endogenous Plk1 and we clearly observed a reduction in cells entering mitosis when Plk1 was depleted (Figure 2E). Reconstitution of LAP-Plk1 rescued recovery, albeit not completely (Figure 2E). However, reconstitution of AKAP-LAP-Plk1 or H2B-LAP-Plk1 was unable to significantly increase the fraction of cells that could recover (Figure 2D). These results indicate that Plk1 function requires free diffusion of Plk1 between nucleus and cytoplasm, not only to be efficiently phosphorylated at T210 but also to be able to promote recovery from a DNA-damage-induced arrest.

### Bora localizes in the cytoplasm

**Figure 3:**
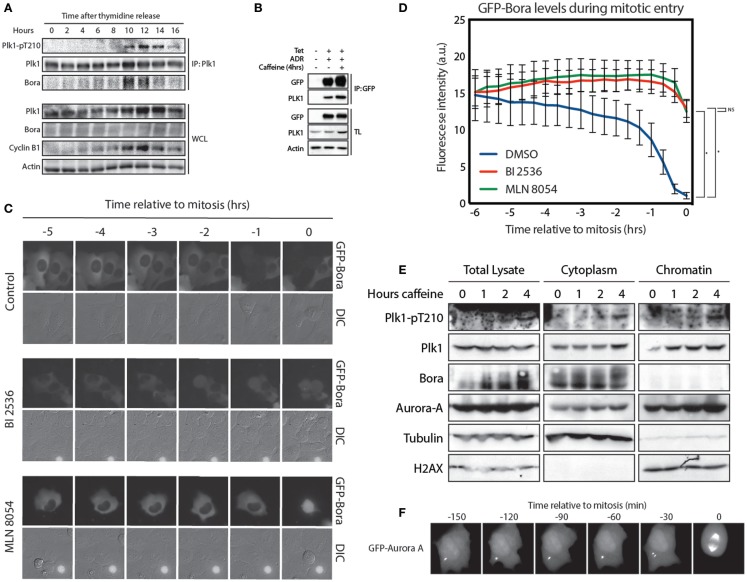
**Bora localizes in the cytoplasm and is degraded 2 h before mitosis**. **(A)** U2OS cells were synchronized at the G1/S border using a 24-h thymidine block and harvested at the indicated times after thymidine release. Plk1 was immunoprecipitated and protein levels were analyzed by western blot. **(B)** Tetracycline inducible U2TR cells stably expressing GFP-Bora were synchronized in G2, DNA damage was induced for 1 h using 0.5 μM adriamycin. Tetracycline was added where indicated and cells were arrested for 16 h. Caffeine was added for 4 h where indicated. Cells were harvested and GFP-Bora was immunoprecipitated. Protein levels were analyzed by western blot. **(C)** Tetracycline inducible U2TR cells stably expressing GFP-Bora were filmed asynchronously and expression was induced by tetracycline. Cells were treated as indicated with 100 nM BI 2536 or 1 μM MLN 8054. Stills from a movie showing DIC- and GFP-images are shown of cells entering mitosis. **(D)** Quantification of 3C, total GFP expression levels were measured over time. Error bars represent SEM of 10 individually quantified cells. *p < 0.0001. **(E)** U2OS cells were arrested for 16 h after induction of DNA damage in G2 with 0.5 μM adriamycin. Recovery was induced with caffeine for the indicated times. Total lysates were obtained as well as cytoplasmic and chromatin fractions using the cell fractionation protocol from Méndez and Stillman (21). **(F)** Stills from real time imaging of a GFP-Aurora A-expressing U2OS cell entering mitosis.

Activation of Plk1 is carried out by Aurora A, which phosphorylates T210 in G2. This phosphorylation event requires the co-factor Bora (11, 12). To further study Plk1/Bora complex formation, we synchronized cells at the G1/S border and performed a time course. We immunoprecipitated Plk1 and analyzed the amount of Bora that co-immunoprecipitated to see when these proteins started to interact. We observed that interaction between Plk1 and Bora occurs already early after thymidine release, possibly reflecting Plk1 functions during replication (27), while phosphorylation of Plk1 at T210 accumulates later in G2 (Figure 3A). Despite our best efforts, we were unable to detect any Aurora A in these co-immunoprecipitation experiments, which may indicate that the interaction of Aurora A with the Plk1-Bora complex might be extremely transient (data not shown). Since available antibodies that recognize Bora are not suitable for immunofluorescence, we were unable to determine the exact localization of endogenous Bora in cells (data not shown). Therefore, we generated a tetracycline-inducible GFP-Bora cell line to study Bora localization (17). Induction with tetracycline resulted in efficient induction of GFP-Bora expression (Figure 3B). In addition, GFP-Bora could efficiently co-immunoprecipitate Plk1 and this interaction increases when recovery is induced by the addition of caffeine (Figure 3B). We next monitored the localization of GFP-Bora. In *Drosophila*, Bora has been shown to initially localize in the nucleus, then transfer to the cytoplasm in early prophase until nuclear envelope breakdown (16). However, we were unable to detect any substantial nuclear signal of GFP-Bora in line with an earlier report (17); instead, we clearly observed that Bora was persistently cytoplasmic throughout interphase. When cells enter mitosis, Bora is targeted for degradation in a Plk1- and βTrCP-dependent manner (17–19). To see if GFP-Bora behaves in a similar manner, we filmed cells expressing GFP-Bora entering mitosis. Indeed, we observed a reduction in GFP-Bora expression approximately 2 h before cells entered mitosis, similar to a recent report (Figures 3C,D) (17). In accordance with the literature, degradation of Bora was abrogated when Plk1 was inhibited. In addition, inhibition of Aurora A had a similar effect on the stability of Bora [Figures 3C,D; (17)], which is consistent with the continuous activation of Plk1 by Aurora-A during mitosis (14).

Because we were unable to monitor endogenous Bora by immunofluorescence, we performed a fractionation assay to separate cytoplasmic proteins from the nuclear proteins (21). We synchronized cells in G2 and induced DNA damage. About 16 h after the DNA damaging insult, we induced recovery by addition of caffeine and harvested cells after 1, 2, and 4 h to monitor T210 phosphorylation. Similar to our observations with the GFP-Bora-expressing cell line, endogenous Bora appeared to be strictly cytoplasmic (Figure 3E). Interestingly, Aurora A was clearly present in both the nucleus and the cytoplasm. Nuclear enrichment of GFP-Aurora-A next to its well-known centrosomal localization during G2 was also observed by monitoring a GFP-Aurora A expressing U2OS cell during mitotic entry (Figure 3F). Although the general idea is that Aurora A localizes predominantly at the centrosomes, nuclear localization has also been reported by overexpression studies as well as on endogenous levels (15, 28). Phosphorylation of Plk1 at T210 did not seem to be preferentially present in the cytoplasm or in the nucleus as the signal appeared in both places at 4 h after caffeine addition. These results, combined with the data presented in Figure 2, suggest that Plk1 is phosphorylated at T210 at the centrosomes from where active Plk1 subsequently can translocate to the nucleus.

## Discussion

Plk1 localization is highly dynamic during the cell cycle. Activation starts in G2, presumably at the centrosomes, but activity monitored by a FRET-based biosensor is first observed in the nucleus approximately 5 h before mitosis (12, 14). Similarly, Plk1 localization to kinetochores, which depends on Plk1 activity, occurs at the S/G2 transition (15). These observations raise questions about the exact location where Plk1 is initially activated.

**Figure 4:**
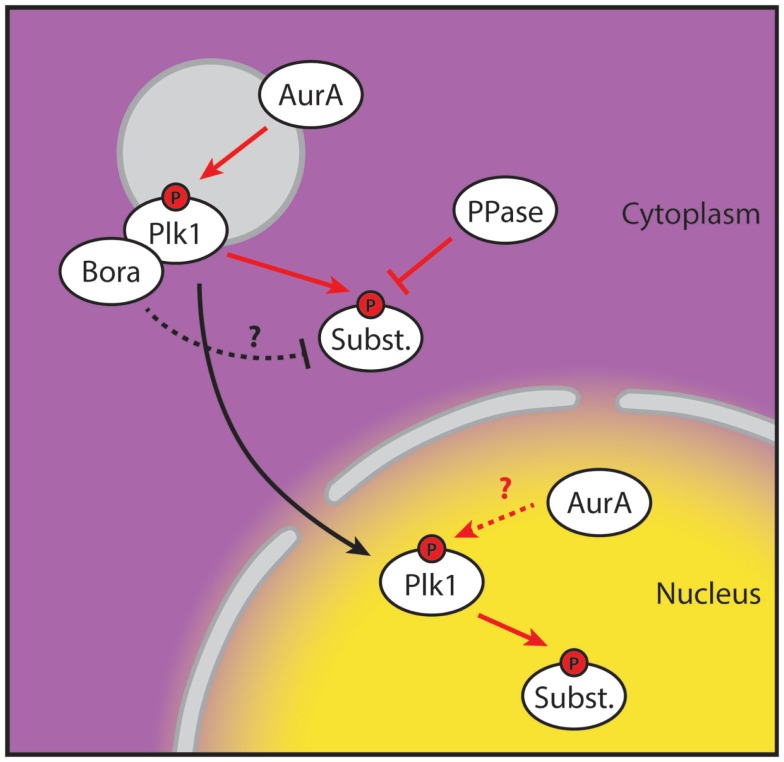
**Spatial separation of Plk1 phosphorylation and activity**. Proposed model for Plk1 activation during G2. Plk1 binds to its co-factor Bora and is subsequently activated at the centrosome on T210 by Aurora A. Activity of the T210-phosphorylated Plk1 is inhibited in the cytoplasm, possibly by Bora or by phosphatase activity directed at its substrates. T210-phosphorylated Plk1 then translocates to the nucleus where the inhibitory factors of Plk1 activity are absent and Plk1 starts phosphorylating its substrates. The contribution of nuclear Aurora A is currently unclear. Yellow and purple indicate high and low levels of Plk1 substrate phosphorylation, respectively. Red arrows indicate phosphorylation events. The “P” indicates phosphorylation of T210.

Here, we provide proof that stable phosphorylation of Plk1 targets first occurs in the nucleus. Since the H2B-tagged and diffusible probes showed similar profiles, we ruled out the possibility that detection of Plk1 activity was affected by diffusion or active import of the phosphorylated FRET-probe from the cytoplasm (Figure 1). This, in combination with our observation that centrosome-tethered Plk1 is phosphorylated at T210 while H2B-tethered Plk1 is not, implies that Plk1 needs to be in the cytoplasm for its initial activation and subsequently move into the nucleus to phosphorylate its targets. Neither the centrosome-tethered variant nor the nuclear-restricted variant of Plk1 is able to rescue recovery from a DNA damage-induced arrest in cells depleted of endogenous Plk1 (Figure 2), further supporting a model in which centrosomal activation is followed by translocation to the nucleus in order for Plk1 to execute its function in regulating mitotic entry. Finally, we show that Bora localizes exclusively in the cytoplasm and its degradation is induced approximately 2 h before cells enter mitosis. Our data suggest that Plk1 is phosphorylated at T210 at the centrosomes but phosphorylation of Plk1 targets is somehow inhibited in the cytoplasm, whereas activated Plk1 that translocates to the nucleus can phosphorylate its targets (Figure 4).

There are several possible explanations for the preferential target phosphorylation by Plk1 in the nucleus. Phosphorylation of the FRET-based biosensor can easily be reversed as Plk1 inhibition decreases the CFP/YFP-ratio to basal levels in approximately half an hour (14). This observation shows that phosphatases are also at play and dephosphorylate the FRET-probe. Thus, preferential substrate phosphorylation by Plk1 in the nucleus could either be due to accumulation of active Plk1 to the nucleus or it could be due to higher phosphatase activity directed toward Plk1 targets in the cytoplasm. Our observation that the relative level of T210-phosphorylation is similar in the cytoplasm compared to the nucleus suggests that preferential substrate phosphorylation in the nucleus is due to high phosphatase activity in the cytoplasm. One of the candidate phosphatases that could suppress the activity of Plk1 toward its cytoplasmic substrates is PP2A/B55 that is highly active in cytoplasm until its inhibition by Greatwall kinase and endosulfine shortly before mitotic entry (29–31). In addition, PP2A/B55 has recently been shown to counteract Plk1 activity through dephosphorylation of T210 after DNA damage (32).

We find that a version of Plk1 that is tethered to the centrosome can be phosphorylated at T210, albeit less than the wild type version of Plk1. However, it is not possible to functionally rescue Plk1 function when Plk1 is tethered to the centrosome, something others have observed as well (33). In addition, forced nuclear localization did not result in phosphorylation at T210 during recovery nor did it result in a functional rescue. These results suggest that dynamic localization of Plk1 during the cell cycle is of utmost importance to carry out its functions. Activity of Plk1 can direct it to different subcellular sites. For instance, Plk1 is recruited in a Cdk-dependent manner to the centrosomes by hCenexin1 (9) and to kinetochores by BubR1, Bub1, or INCENP (8, 34, 35). In addition, Plk1 can also create its own docking site to target itself to the kinetochores through PBIP1 (5) or mediate its translocation to the central spindle in anaphase through PRC1 (7). These reports and our current results are clear indications that dynamic localization of Plk1 is indispensible for proper execution of its functions during G2 and mitosis.

Phosphorylation of Plk1 at T210 by Aurora A requires Bora (11, 12). Our data strengthen the idea that initial phosphorylation occurs at the centrosomes as we observe that Bora is strictly cytoplasmic as opposed to phosphorylation in the nucleus. Bora binds to the Plk1 Polo-box domain in a Cdk-dependent manner (18). This not only allows Aurora A to phosphorylate Plk1 at T210 but additionally targets Bora itself as a substrate of Plk1 (18, 19), since phosphorylation by Plk1 targets Bora for βTrCP-dependent proteasomal degradation. While the literature so far has suggested that this takes place in mitosis, we observed that Bora levels already diminish at 2 h before cells enter mitosis (17). This observation coincides roughly with the time that substrate phosphorylation by Plk1 is first observed in the cytoplasm. Since Bora most likely interacts with Plk1 in the cytoplasm, it is tempting to speculate that despite the T210-phosphorylation that occurs in the cytoplasm, Plk1 target phosphorylation is somehow inhibited until Plk1 translocates to the nucleus where it cannot bind Bora anymore (Figure 4). This could either take place by direct inhibition of phosphorylation by Plk1, or as mentioned earlier, by rapid reversion of phosphorylation of Plk1 targets by a phosphatase that is enriched in the cytoplasm. As the degradation of Bora before mitotic entry coincides with Plk1 target phosphorylation in the cytoplasm, it is a distinct possibility that Bora can function as an inhibitor of Plk1 activity, at least toward its other substrates, in the cytoplasm. It would therefore be interesting to test if binding of Bora to Plk1 can prevent efficient binding to its other substrates, for example by occupying the Polo box domain binding site. Further analysis of the Bora-Plk1 complex and the effect of Bora-binding to Plk1-dependent phosphorylation of other Plk1 targets will be required to answer these questions.

In addition to the downstream regulation, upstream regulation of Aurora A activity during G2 and how this relates to specific timing of T210-phosphorylation on Plk1 is currently unclear. Aurora A relies on several co-factors to exert its functions and we have previously shown that the Aurora A co-factor TPX2 does not contribute to the activation of Plk1 during G2 and mitosis (14). However, it will be interesting to investigate other Aurora A co-activators and recruiters such as Ajuba (36) or CEP192 (37) and study their impact on timely activation of Plk1. More detailed analysis of timing and activation events of Plk1, Bora, and Aurora A will be required to elucidate the complex spatiotemporal regulation of Plk1 activation during G2.

## Conflict of Interest Statement

The authors declare that the research was conducted in the absence of any commercial or financial relationships that could be construed as a potential conflict of interest.

## References

[1] Bruinsma W Raaijmakers JA Medema RH. Switching Polo-like kinase-1 on and off in time and space. Trends Biochem Sci (2012) 37:534–42. 10.1016/j.tibs.2012.09.005 23141205

[2] Petronczki M Glotzer M Kraut N Peters J-M. Polo-like kinase 1 triggers the initiation of cytokinesis in human cells by promoting recruitment of the rhogef ect2 to the central spindle. Dev Cell (2007) 12:713–25. 10.1016/j.devcel.2007.03.013 17488623

[3] Elia AEH Cantley LC Yaffe MB. Proteomic screen finds pSer/pThr-binding domain localizing Plk1 to mitotic substrates. Science (2003) 299:1228–31. 10.1126/science.1079079 12595692

[4] Elia AEH Rellos P Haire LF Chao JW Ivins FJ Hoepker K The molecular basis for phosphodependent substrate targeting and regulation of Plks by the Polo-box domain. Cell (2003) 115:83–95. 10.1016/S0092-8674(03)00725-6 14532005

[5] Kang YH Park J-E Yu L-R Soung N-K Yun S-M Bang JK Self-regulated Plk1 recruitment to kinetochores by the Plk1-PBIP1 interaction is critical for proper chromosome segregation. Mol Cell (2006) 24:409–22. 10.1016/j.molcel.2006.10.016 17081991

[6] Leung GC Hudson JW Kozarova A Davidson A Dennis JW Sicheri F. The Sak Polo-box comprises a structural domain sufficient for mitotic subcellular localization. Nat Struct Biol (2002) 9:719–24. 10.1038/nsb848 12352953

[7] Neef R Gruneberg U Kopajtich R Li X Nigg EA Sillje H Choice of Plk1 docking partners during mitosis and cytokinesis is controlled by the activation state of Cdk1. Nat Cell Biol (2007) 9:436–44. 10.1038/ncb1557 17351640

[8] Qi W Tang Z Yu H. Phosphorylation- and Polo-box-dependent binding of Plk1 to Bub1 is required for the kinetochore localization of Plk1. Mol Biol Cell (2006) 17:3705–16. 10.1091/mbc.E06-03-0240 16760428 PMC1525235

[9] Soung N-K Park J-E Yu L-R Lee KH Lee J-M Bang JK Plk1-dependent and -independent roles of an ODF2 splice variant, hCenexin1, at the centrosome of somatic cells. Dev Cell (2009) 16:539–50. 10.1016/j.devcel.2009.02.004 19386263 PMC2741019

[10] Jang Y-J Ma S Terada Y Erikson RL. Phosphorylation of threonine 210 and the role of serine 137 in the regulation of mammalian polo-like kinase. J Biol Chem (2002) 277:44115–20. 10.1074/jbc.M202172200 12207013

[11] Seki A Coppinger JA Jang C-Y Yates JR Fang G. Bora and the kinase aurora a cooperatively activate the kinase Plk1 and control mitotic entry. Science (2008) 320:1655–8. 10.1126/science.1157425 18566290 PMC2834883

[12] Macurek L Lindqvist A Lim D Lampson MA Klompmaker R Freire R Polo-like kinase-1 is activated by aurora A to promote checkpoint recovery. Nature (2008) 455:119–23. 10.1038/nature07185 18615013

[13] Barr AR Gergely F. Aurora-A: the maker and breaker of spindle poles. J Cell Sci (2007) 120(Pt 17):2987–96. 10.1242/jcs.013136 17715155

[14] Bruinsma W Macurek L Freire R Lindqvist A Medema RH. Bora and Aurora-A continue to activate Plk1 in mitosis. J Cell Sci (2014) 127(Pt 4):801–11. 10.1242/jcs.137216 24338364

[15] Akopyan K Silva Cascales H Hukasova E Saurin AT Müllers E Jaiswal H Assessing kinetics from fixed cells reveals activation of the mitotic entry network at the S/G2 transition. Mol Cell (2014) 53:843–53. 10.1016/j.molcel.2014.01.031 24582498

[16] Hutterer A Berdnik D Wirtz-Peitz F Zigman M Schleiffer A Knoblich JA. Mitotic activation of the kinase aurora-A requires its binding partner Bora. Dev Cell (2006) 11:147–57. 10.1016/j.devcel.2006.06.002 16890155

[17] Feine O Hukasova E Bruinsma W Freire R Fainsod A Gannon J Phosphorylation-mediated stabilization of Bora in mitosis coordinates Plx1/Plk1 and Cdk1 oscillations. Cell Cycle (2014) 13(11):1727–36. 10.4161/cc.28630 24675888 PMC4111719

[18] Chan EHY Santamaria A Sillje HHW Nigg EA. Plk1 regulates mitotic aurora A function through betaTrCP-dependent degradation of hBora. Chromosoma (2008) 117:457–69. 10.1007/s00412-008-0165-5 18521620 PMC2921497

[19] Seki A Coppinger JA Du H Jang C-Y Yates JR Fang G. Plk1- and beta-TrCP-dependent degradation of Bora controls mitotic progression. J Cell Biol (2008) 181:65–78. 10.1083/jcb.200712027 18378770 PMC2287288

[20] Hukasova E Silva Cascales H Kumar SR Lindqvist A. Monitoring kinase and phosphatase activities through the cell cycle by ratiometric FRET. J Vis Exp (2012) (59):e3410. 10.3791/3410 22314640 PMC3462574

[21] Méndez J Stillman B. Chromatin association of human origin recognition complex, cdc6, and minichromosome maintenance proteins during the cell cycle: assembly of prereplication complexes in late mitosis. Mol Cell Biol (2000) 20(22):8602–12. 10.1128/MCB.20.22.8602-8612.2000 11046155 PMC102165

[22] Lénárt P Petronczki M Steegmaier M Di Fiore B Lipp JJ Hoffmann M The small-molecule inhibitor BI 2536 reveals novel insights into mitotic roles of polo-like kinase 1. Curr Biol (2007) 17:304–15. 10.1016/j.cub.2006.12.046 17291761

[23] Manfredi MG Ecsedy JA Meetze KA Balani SK Burenkova O Chen W Antitumor activity of MLN8054, an orally active small-molecule inhibitor of aurora A kinase. Proc Natl Acad Sci U S A (2007) 104:4106–11. 10.1073/pnas.0608798104 17360485 PMC1820716

[24] Cheeseman IM Desai A. A combined approach for the localization and tandem affinity purification of protein complexes from metazoans. Sci STKE (2005) 2005:l1. 10.1126/stke.2662005pl1 15644491

[25] Gillingham AK Munro S. The PACT domain, a conserved centrosomal targeting motif in the coiled-coil proteins AKAP450 and pericentrin. EMBO Rep (2000) 1:524–9. 10.1093/embo-reports/kvd105 11263498 PMC1083777

[26] van Vugt MATM Brás A Medema RH. Polo-like kinase-1 controls recovery from a G2 DNA damage-induced arrest in mammalian cells. Mol Cell (2004) 15:799–811. 10.1016/j.molcel.2004.07.015 15350223

[27] Takaki T Trenz K Costanzo V Petronczki M. Polo-like kinase 1 reaches beyond mitosis – cytokinesis, DNA damage response, and development. Curr Opin Cell Biol (2008) 20:650–60. 10.1016/j.ceb.2008.10.005 19000759

[28] Rannou Y Troadec M-B Petretti C Hans F Duterte S Dimitrov S Localization of aurora A and aurora B kinases during interphase. Cell Cycle (2008) 7:3012–20. 10.4161/cc.7.19.6718 18802402 PMC3325910

[29] Alvarez-Fernández M Sánchez-Martínez R Sanz-Castillo B Gan PP Sanz-Flores M Trakala M Greatwall is essential to prevent mitotic collapse after nuclear envelope breakdown in mammals. Proc Natl Acad Sci U S A (2013) 110:17374–9. 10.1073/pnas.1310745110 24101512 PMC3808628

[30] Wang P Galan JA Normandin K Bonneil É Hickson GR Roux PP Cell cycle regulation of greatwall kinase nuclear localization facilitates mitotic progression. J Cell Biol (2013) 202:277–93. 10.1083/jcb.201211141 23857770 PMC3718974

[31] Mochida S Maslen SL Skehel M Hunt T. Greatwall phosphorylates an inhibitor of protein phosphatase 2A that is essential for mitosis. Science (2010) 330:1670–3. 10.1126/science.1195689 21164013

[32] Wang L Guo Q Fisher LA Liu D Peng A Regulation of polo-like kinase 1 by DNA damage and PP2A/B55α. Cell Cycle (2015) 14:157–66. 10.4161/15384101.2014.986392 25483054 PMC4615057

[33] Kishi K van Vugt MATM Okamoto K-I Hayashi Y Yaffe MB. Functional dynamics of Polo-like kinase 1 at the centrosome. Mol Cell Biol (2009) 29:3134–50. 10.1128/MCB.01663-08 19307309 PMC2682011

[34] Goto H Kiyono T Tomono Y Kawajiri A Urano T Furukawa K Complex formation of Plk1 and INCENP required for metaphase–anaphase transition. Nat Cell Biol (2005) 8:180–7. 10.1038/ncb1350 16378098

[35] Elowe S Hümmer S Uldschmid A Li X Nigg EA. Tension-sensitive Plk1 phosphorylation on BubR1 regulates the stability of kinetochore microtubule interactions. Genes Dev (2007) 21:2205–19. 10.1101/gad.436007 17785528 PMC1950859

[36] Hirota T Kunitoku N Sasayama T Marumoto T Zhang D Nitta M Aurora-A and an interacting activator, the LIM protein Ajuba, are required for mitotic commitment in human cells. Cell (2003) 114:585–98. 10.1016/S0092-8674(03)00642-1 13678582

[37] Joukov V Walter JC De Nicolo A. The Cep192-Organized Aurora A-Plk1 cascade is essential for centrosome cycle and bipolar spindle assembly. Mol Cell (2014) 55:578–91. 10.1016/j.molcel.2014.06.016 25042804 PMC4245277

